# Effects of *Nigella sativa* Oil Fractions on Reactive Oxygen Species and Chemokine Expression in Airway Smooth Muscle Cells

**DOI:** 10.3390/plants12112171

**Published:** 2023-05-30

**Authors:** Asma Mosbah, Hanane Khither, Camélia Mosbah, Abdelkader Slimani, Abdelkader Mahrouk, Salah Akkal, Gema Nieto

**Affiliations:** 1 Laboratory of Applied Biochemistry, Faculty of Natural and Life Sciences, University Constantine 1, Constantine 25000, Algeria; mahroukabk@gmail.com; 2 Laboratory of Applied Biochemistry, Faculty of Natural and Life Sciences, University of Ferhat Abbas Setif 1, Setif 19000, Algeria; h.khither@yahoo.fr; 3 Laboratory of Natural Substances, Bioactive Molecules and Biotechnological Applications, Larbi Ben M’hidi University, Oum El Bouagui 04000, Algeria; kamimosbah@yahoo.fr; 4 Unit of the Valorization of Natural Resources, Bioactive Molecules and Physicochemical and Biological Analysis, Faculty of Exact Sciences, University Constantine 1, Constantine 25000, Algeria; slimani.25000@gmail.com (A.S.); salah4dz@yahoo.fr (S.A.); 5 Department of Food Technology, Food Science and Nutrition, Faculty of Veterinary Sciences, Regional Campus of International Excellence “Campus Mare Nostrum”, University of Murcia, Espinardo, 30071 Murcia, Spain

**Keywords:** airway smooth muscle cells, asthma, chemokines, inflammation, *Nigella sativa* oil, reactive oxygen species

## Abstract

Background: many previous studies have demonstrated the therapeutic potential of *N. sativa* total oil fractions, neutral lipids (NLs), glycolipids (GLs), phospholipids (PLs), and unsaponifiable (IS) in asthma patients. We therefore tested its effect on airway smooth muscle (ASM) cells by observing its ability to regulate the production of glucocorticoid (GC)-insensitive chemokines in cells treated with TNF-α/IFN-γ, and its antioxidative and reactive oxygen species (ROS) scavenging properties. Materials and methods: the cytotoxicity of *N. sativa* oil fractions was assessed using an MTT assay. ASM cells were treated with TNF-α/IFN-γ for 24 h in the presence of different concentrations of *N. sativa* oil fractions. An ELISA assay was used to determine the effect of *N. sativa* oil fractions on chemokine production (CCL5, CXCL-10, and CXCL-8). The scavenging effect of *N. sativa* oil fractions was evaluated on three reactive oxygen species (ROS), O_2_•^−^, OH•, and H_2_O_2_. Results: our results show that different *N. sativa* oil fractions used at 25 and 50 µg/mL did not affect cell viability. All fractions of *N. sativa* oil inhibited chemokines in a concentration-dependent manner. Interestingly, the total oil fraction showed the most significant effect of chemokine inhibition, and had the highest percentage of ROS scavenging effect. Conclusion: these results suggest that *N. sativa* oil modulates the proinflammatory actions of human ASM cells by inhibiting the production of GC-insensitive chemokines.

## 1. Introduction

Asthma is a chronic inflammatory condition of the respiratory system (airways) characterized by airway inflammation, intermittent airflow obstruction, and bronchial hyper-responsiveness. Approximately 5 to 10% of patients with severe asthma are poorly responsive to high doses of glucocorticoids (GCs) (oral or inhaled) [1]. Airway inflammation plays a pivotal role in asthma severity by promoting excessive contraction of the airway smooth muscle (ASM) and remodeling of the airways, both contributing to an acute asthma attack. ASM may also participate in progression/perpetuation of the airway inflammation via its ability to produce a large array of cytokines, growth factors, and chemokines, which are capable of attracting different immune cells, including T cells, eosinophils, and neutrophils [2]. ASM has been also implicated in the poor response of severe asthma patients to GCs by being refractory to their anti-inflammatory action [3]. It was suggested that development of GC insensitivity in ASM cells could result from the concerted action of different inflammatory cytokines. Indeed, treatment of ASM cells with both TNF-α and IFN-γ stimulated the expression of a number of proasthmatic proteins (cytokines, adhesion molecules, transcription factors, etc.) that were clearly insensitive to fluticasone [3]. The underling mechanisms of GC insensitivity involved the over-activation of the transcription factor IRF-1, expression of dominant negative GRβ, and the protein phosphatase PP5 [3]. Collectively, these observations suggest that targeting these proinflammatory and GC-insensitive pathways in ASM cells may offer new therapeutic avenues for asthma.

GC insensitivity may also derive from reactive oxygen species (ROS). Inflammatory cells, such as T cells, helper T cells, mast cells, macrophages, basophils, and eosinophils, which are involved in airway inflammation in asthma [4], produce different reactive oxygen species [5]. ROS have been involved in the pathogenesis of asthma as evidenced by the increased markers of oxidative stress in various lung specimens including sputum, lungs, exhaled breath, and blood, which often correlate with disease severity or poor control [6,7]. Oxidative stress may contribute to the progression and/or aggravation of asthma via multiple mechanisms including alteration of the epithelium or ASM cells, leading to proremodeling and proinflammatory actions [6]. Indeed, oxidative stress stimulates the production of various proinflammatory mediators, chemokines, adhesion molecules, and eosinophil granule proteins [7,8,9,10]. Evidence suggests that ROS may promote the development of GC insensitivity by interfering with GC receptor (GRα) signaling pathways, including inhibition of histone deacetylase 2 (HDAC2) activity and/or interference with GRα nuclear trafficking [11].

*Nigella sativa* (*N. sativa*) is an herbaceous plant, native to Southern Europe, North Africa and Southeast Asia [12,13,14]. This plant is traditionally used worldwide for culinary and medicinal purposes [15,16]. *N. Sativa* has been shown to have numerous properties, including immune-stimulatory, anti-inflammatory, hypoglycemic, antihypertensive, antimicrobial, antiparasitic, antioxidant, and anticancer actions [17,18,19,20]. *N. sativa* and its constituents have been traditionally used to relieve respiratory disorders such as asthma, bronchospasm, and chest congestion, although the precise mechanisms of action have not been elucidated [21]. Few studies have examined whether *N. sativa* has antiasthma properties. Indeed, it was reported that *N. sativa* supplementation improved asthma control, reduction in exacerbations, inflammatory markers such as IFN-γ, and blood eosinophils [22,23]. *N. sativa* also exerts immunomodulatory and therapeutic effects in patients with allergic diseases (allergic rhinitis, bronchial asthma, and atopic eczema), possibly via the inhibition of proinflammatory cytokines, downregulating PGD_2_ and COX-2 expressions, and decrease of airway inflammatory cell infiltration [24,25]. In vitro studies reported the beneficial role of carvacrol, one of the key components of *N. sativa*, in guinea pig tracheal preparations. Carvacrol was reported to exert broncho relaxant and functionally antagonistic effects on muscarinic receptors [26], histamine (H1) receptors [27], an inhibitory effect on calcium [28], and an opening effect on potassium channels [29]. A boiled extract of *N. sativa* indicated a bronchodilatory effect on asthmatic patients by increasing pulmonary function [30].

To manage patients with severe asthma, the chronic administration of high doses of GC is required, which is often associated with serious adverse effects. In corticosteroid-resistant asthma, the need exists for novel therapies. Clinical trials are oriented toward natural biomolecules extracted from medicinal plants for the treatment of various diseases. In this study, we provide the first evidence that *N. sativa* and its fractions, neutral lipids (NLs), glycolipids (GLs), phospholipids (PLs), and unsaponifiable (IS), could exert therapeutic effects in asthma by inhibiting the production of GC-insensitive chemokines in human ASM cells, a clinically relevant cell model.

## 2. Results

### 2.1. Extraction Yield

The total oil and its fraction yields are summarized in Table 1.

### 2.2. Analysis of N. sativa Total Oil Lipid Components Using Gas Chromatography–Mass Spectrophotometer (GC–MS)

**Figure 1:**
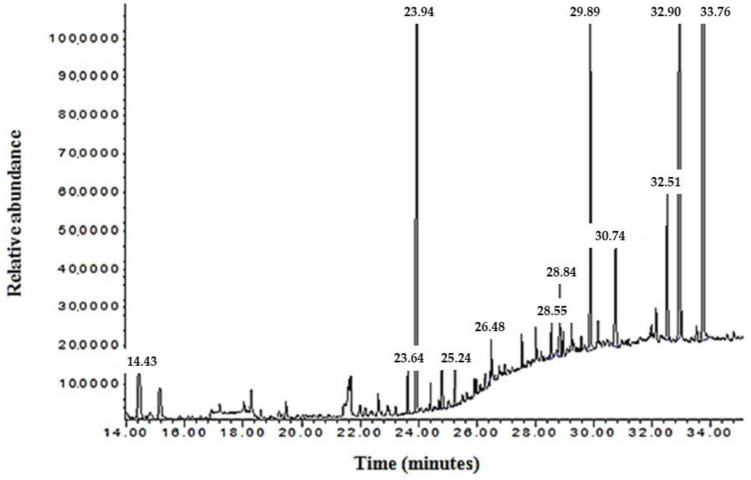
Gas chromatography–mass spectrometry (GC–MS)chromatogram of fatty acid compounds in *N. sativa* total oil (TO).

The total oil of *N. sativa* was analyzed using GC–MS. The obtained chromatogram exhibited multiple peaks at various retention times and intensities (Figure 1).

Compounds corresponding to these peaks were identified based on their molecular weights. The chemical compositions of *N. sativa* TO identified by GC–MS are listed in Table 2. In total, 18 constituents were identified in the TO fraction obtained from Algerian *N. sativa* seeds using the GC–MS technique. 

To determine the efficiency of *N. sativa* oil fractions, antiradical, anti-inflammatory, and cell viability assays were performed. The half-maximal inhibitory concentration (IC_50_) values of *N. sativa* oil fractions were determined.

### 2.3. Antiradical Assays

**Figure 2:**
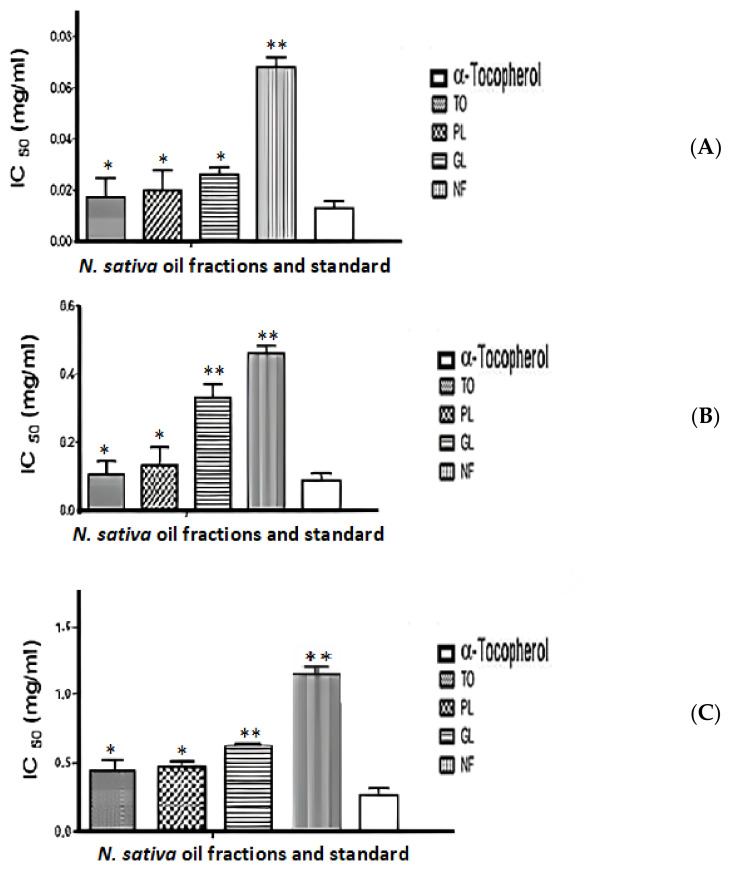
Scavenging effect of total oil (TO), phospholipids (PLs), glycolipids (GLs), and neutral fraction (NF) (**A**) superoxide anion radical (O_2_•^−^), (**B**) hydroxyl radical (OH•), and (**C**) hydrogen peroxide (H_2_O_2_). α-Tocopherol was used as positive control. Values were expressed as the mean ± SD of experiments performed in triplicate, * *p* < 0.05, ** *p* < 0.001.

Antiradical properties of *N. sativa* oil fractions were compared using different reactive oxygen species (O_2_•^−^, OH•, and H_2_O_2_). All assays showed a similar trend in the scavenging of free radicals. Figure 2 indicates that TO has the highest scavenging effect, followed by PL, GL, and NL. However, this scavenging effect remained lower than that of positive control α-tocopherol.

For superoxide anion radical, total oil (TO) presents the best scavenging activity, with an IC_50_ of 0.0172 mg/mL, followed by phospholipids (PLs), glycolipids (GLs), and neutral lipids (NFs) fractions, with an IC_50_ of 0.0197, 0.0261, and 0.0680 mg/mL, respectively. The same pattern was also reported for hydroxyl radical and H_2_O_2_ scavenging capacity, when TO showed the highest antiradical activity with an IC_50_ of 0.105, at the concentration of 0.440 mg/mL, followed by PL 0.132, at the concentration of 0.465 mg/mL, GL 0.330, at the concentration of 0.626 mg/mL and, NF 0.460, at 0.930 mg/mL, respectively.

### 2.4. MTT Assay

To investigate the effect of *N. sativa* oil fractions on cell viability, ASM cells were treated with 25 and 50 µg/mL *N. Sativa* TO fractions for 24 h and 48 h, and subjected to the MTT colorimetric assay. 

**Figure 3:**
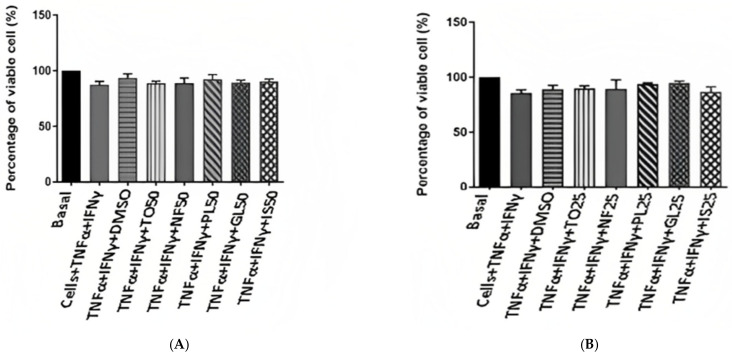
Cell proliferation (MTT assay) results of airway smooth muscle (ASM) cells following treatment with *N. sativa* oil fractions at concentrations of (50 µg/mL) (**A**) and (25 µg/mL) (**B**) for 48 h of incubation. All values are expressed as mean ± SEM from two different replicates.

The percent cell viability of ASM cells against *N. sativa* oil fractions, as observed by the MTT assay, is presented in Figure 3. The results show that all fractions of *N. sativa* oil demonstrate no toxic activity on ASM cells at the used dosages and treatment periods.

### 2.5. Anti-Inflammatory Activity of N. sativa Oil Fractions

In the present study, we aimed to investigate the effect of *N. sativa* oil fractions on the expression of chemokines in ASM cells treated with TNF-α and IFN-γ.

**Figure 4:**
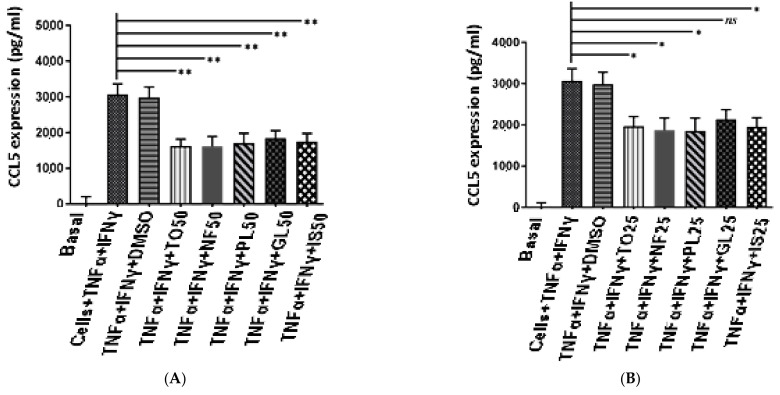

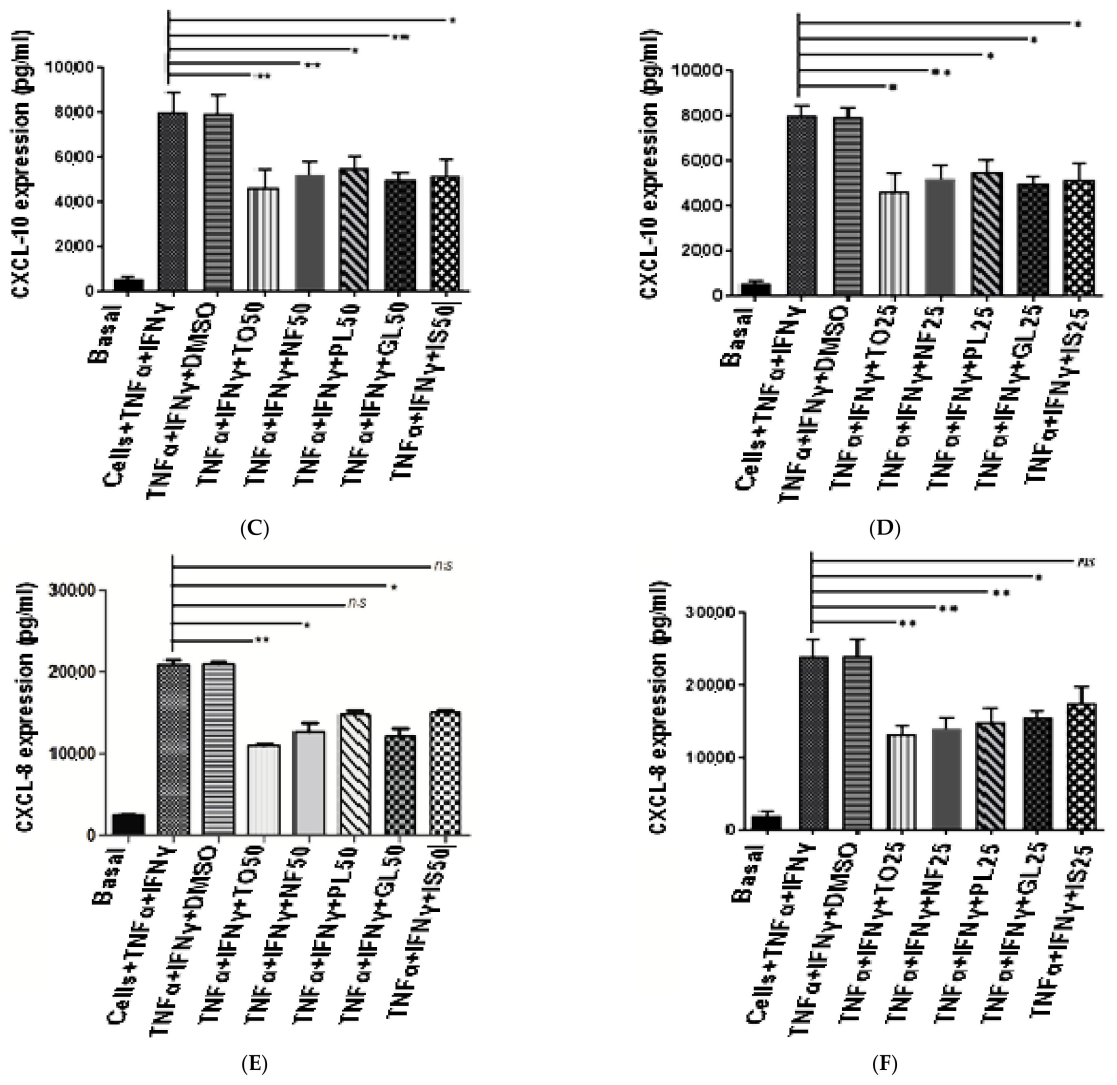
*Nigella sativa* oil fractions differentially regulate chemokine secretion in airway smooth muscle (ASM) cells stimulated for 24 h with TNF-α (10 ng/mL) and IFN-γ (25 ng/mL) compared to unstimulated ASM cells (the basal). Levels of CCL5 (**A**,**B**), CXCL-10 (**C**,**D**), and CXCL-8 (**E**,**F**) were analyzed as described in the Materials and Methods section. Results shown as mean ± SEM of *n* = 6 different donors. TO: total oil, NF: neutral fraction, GL: glycolipids, PL: phospholipids, IS: unsaponifiable * *p* < 0.05, ** *p* < 0.001, ns = not significant.

As shown in Figure 4, both TNF-α and IFN-γ increased chemokine production in ASM cells from subjects with asthma compared to unstimulated ASM cells (the basal). Interestingly, the different *N. sativa* fractions induced a dose-dependent inhibition of chemokine production, with the highest inhibition achieved at 50 µg/mL for all fractions. In ASM-treated cells, TO Inhibited CCL5 secretion by 43.33%, CXCL-10 secretion by 40.03%, and CXCL-8 secretion by 47.62% (*n* = 6). The NF fraction inhibited CCL5 secretion by 41.66%, CXCL-10 secretion by 36.25 %, and CXCL-8 secretion by 39.53%. The PL fraction inhibited CCL5 secretion by 40.05%, CXCL-10 secretion by 31.25%, and CXCL-8 secretion by 29.48%. The GL fraction inhibited CCL5 secretion by 36.33%, CXCL-10 secretion by 37.50%, and CXCL-8 secretion by 42.26%. IS inhibited CCL5 secretion by 38.02%, CXCL-10 secretion by 35.62%, and CXCL-8 secretion by 28.11% (Figure 4).

## 3. Discussion

The present study aimed to explore the anti-inflammatory effect of different fractions of *N. sativa* oil in terms of chemokine secretion in human ASM cells. This study found that *N. sativa* TO and derived fractions suppressed the production of GC-insensitive chemokine action by human ASM cells, and possess ROS scavenging activity. This study shows the potential benefit of *N. sativa* by acting on ASM cells, a key player in the pathogenesis of asthma.

The chemical composition of *N. sativa* TO identified by GC–MS indicated the presence of linoleic acid (18:2n-6), oleic acid (18:1n-9), palmitic acid (16:0), and stearic acid based on comparative value for the standards present in the database. Comparing our results with those reported by Ramadan et al. (2003) [31], which used Egyptian *N. sativa*, indicated that linoleic acid, oleic acid, and palmitic acid measured for Egyptian TO were higher than that of Algerian TO. Another study showed that the concentrations of fatty acids (oleic, linoleic, palmitic, and stearic acids) in Algerian *N. sativa* oil were higher than those of Indian and Ethiopian plants [32] (Table 3). These differences with regard to the qualitative and quantitative aspects of *N. sativa* could be explained by the genetic properties of seeds, geographical origin, storage conditions, harvest duration, and extraction methods. 

The present report also confirmed the radical scavenging property of the different *N. sativa* oil fractions by showing their ability to scavenge O_2_•^−^, OH•, and H_2_O_2_ in a dose-dependent manner. The total oil (TO) showed the highest scavenging capacity in all assays, followed by phospholipids (PLs), glycolipids (GLs), and neutral fraction (NFs). Our results of the antiradical activities are in agreement with previous studies showing that TO and PLs presented a potent source of antioxidant compounds, which may explain the therapeutic effect exerted by these fractions [33,34]. The characterization by GC–MS showed the existence of many antioxidant fatty acids, such as linoleic, oleic, palmitic, and stearic acids, and 14 other hydrocarbons. The antioxidant activity of TO and different polar and apolar fractions could be explained by the phytochemical analysis due to the presence of polyphenols, diene functional groups, and unsaturated fatty acids, which are known for their antioxidant activity [31]. Palmitic, oleic, and linoleic acids have been reported to exert a potent antioxidant effect in different assays [35,36,37]. Oxidative stress is involved in the pathophysiology of many human diseases, including asthma. The evidence showed that both increased ROS production by inflammatory cells infiltrated within the airways and/or impairment of the antioxidant responses could contribute to asthma severity and exacerbations [38,39,40]. The inflammatory answer is an important factor to the exacerbation of ROS production in organ injury. The study conducted by Fusco et al. (2020) showed increased plasma levels of proinflammatory cytokines, particularly TNF-α, IL6, and IL-1β, after intestinal ischemia/reperfusion injury [41].

A number of previous studies have shown the therapeutic benefit of *N. sativa* administered via different routes (i.p., orally) in experimental models of asthma (rat, mouse) sensitized and challenged with ovalbumin [42]. *N. sativa* was shown to reduce allergen-induced airway inflammatory markers (Th2 cytokines, white blood cells) and lung histological changes. Our present report shows that these beneficial effects could result from the radical scavenging activity of *N. sativa.*

ASM has been considered as an important player in the pathogenesis of asthma via its contribution to airway remodeling, abnormal lung function, and airway inflammation [43,44]. Indeed, ASM cells can secrete a variety of inflammatory mediators, including chemokines, which could regulate airway inflammation via the recruitment and activation of various key inflammatory cells, monocytes, eosinophils, and T cells [45]. In cultured ASM cells, a combination of both TNF-α and IFN-γ leads to the synergistic production of various proinflammatory chemokines (CXCL-8, CCL-11, CX3CL-1, CCL5, and CXCL-10, among others) [30,46]. In addition, chemokine production by TNF-α and IFN-γ has been shown to be insensitive to GCs via multiple mechanisms [3], thus representing a unique cellular model to dissect the mechanisms of GC insensitivity in asthma, or find new strategies to reverse it. We made the novel finding that *N. sativa* and its fractions inhibited the production of GC-insensitive chemokines (CCL5, CXCL-8, and CXCL-10) induced by TNF-α and IFN-γ in ASM cells from both healthy and asthmatic patients. Although we have not dissected the underlying mechanisms by which *N. sativa* suppressed the production of inflammatory chemokines, it is possible that *N. sativa* interfered with the function of key transcription factors such as NF-κB, IRF-1, and STATs. Additional studies are clearly needed to address these possibilities. These inhibitory actions were not due to any cytotoxic effect as assessed by the MTT assay. Together, our results suggest that the anti-inflammatory actions of *N. sativa*, seen in various animal models of allergic asthma [42], may derive from their capacity to suppress chemokine production from ASM cells.

Previous studies have confirmed the ability of *N. sativa* and its fractions to exert therapeutic action using isolated airway preparations from asthmatic patients [30]. Essential oil of *N. sativa* exhibited an inhibitory effect on the cyclooxygenase and 5-lipoxygenase pathways of arachidonic acid metabolism and membrane lipid peroxidation. Similarly, Boskabady et al. (2008) showed that different *N. sativa* fractions (n-hexane, dichloromethane, methanol, and aqueous) can relax isolated tracheal ring preparations, and the most potent relaxant effect was detected for methanol and dichloromethane fractions [47]. The therapeutic effect of *N. sativa* on airway preparations may result from a direct action on ASM cells via several mechanisms, including (1) interfering with calcium channel blocking activity [28], (2) modulation of potassium channels [29], (3) inhibition of muscarinic receptor function [26], (4) histaminic antagonistic activity receptor [27], and (5) activation of β2-adrenoceptors [48]. Together, these results strongly support a therapeutic action of *N. sativa* and its fractions in asthma via their action on ASM cells.

Herbal extracts showed complex mechanisms of action compared with conventional drugs, which may involve proinflammatory cytokine secretion through various molecular signaling pathways. Several mechanisms of action have been suggested for *N. sativa* extracts. In the present study, the inhibition of the production of GC-insensitive chemokines (CCL5, CXCL-8, and CXCL-10) induced by TNF-α and IFN-γ may involve the mechanism of Th1/Th2 cytokine modulation, which is attributed to the inhibition of the signal transducer and activator of transcription-6 (STAT6) signaling pathway [49,50].

It is important to mention some of the limitations of the present study. First, we have not provided any mechanisms to explain the anti-inflammatory actions of *N. sativa* or its fractions. Looking at the activity of different cytokine-associated transcriptions may address this issue. We have also limited our study at the preventive action of *N. sativa* (2 h preincubation prior cytokines), and it is not known whether the in vitro beneficial action of *N. sativa* would still be seen if used therapeutically (after cytokines). Finally, it would also be interesting to repeat these experiments in ASM cells obtained from nonsevere asthma patients, which exhibit an abnormal phenotype including a constitutive state of GC insensitivity [51].

## 4. Materials and Methods

### 4.1. Materials

All solvents, chemical compounds (fatty acids, MTT, xanthine), and biological compounds (TNF-α, IFN-γ, XOR) were purchased from Sigma-Aldrich (Darmstadt, Germany). Commercial organic solvents methanol, hexane, chloroform, acetone, and DMSO were of analytical grade (∼99.5%). ELISA kits were performed using R&D System Duoset kits (Minneapolis, MN, USA).

### 4.2. Collection of the N. sativa Plant

Seeds of *N. sativa* used in this study were collected from Algerian Sahara, Bechar in 2016. The plant was identified by Dr. Houssine Laouar from Setif 1 University, Algeria. The specimen was deposited at the Natural Biological Resources Development laboratory at Setif 1 University, Algeria. Seeds were cleaned with water, dried, and stored in darkness at 4 °C until use.

### 4.3. Human Airway Smooth Muscle (ASM) Cell Culture

Primary human ASM cells were obtained from consenting healthy subjects and asthmatic patients (Glenfield Hospital patients and staff 6). ASM cells were isolated from endobronchial biopsies as described previously [52,53,54,55]. Written informed consent was gained from all participants prior to their involvement.

This study was approved by the Leicestershire, Northamptonshire, and Rutland Research Ethics Committee (references: 4977, 04/Q2502/74, and 08/H0406/189).

All experiments involving human ASM cells were performed in Dr Amrani’s laboratory at the University of Leicester.

### 4.4. Preparation of Extracts

*N. sativa* seed powder (30 g) was extracted in methanol solvent (400 mL) using a Soxhlet extractor for two hours at room temperature (27 °C). Methanol was evaporated at reduced pressure at 40 °C using a rotary evaporator (BÜCHI 461). The resulting extract was mixed with 200 mL of hexane solvent in a bulb for settling, until the formation of two distinct phases: methanol (green color) and hexane (yellow color). The latter phase was recovered, and hexane solvent was evaporated at 40 °C to obtain total oil (TO), characterized by a greenish color.

The extracted oil fraction was then applied on a silica gel column to be separated into the neutral lipids fraction (NF), glycolipids (GLs), phospholipids (PLs), and unsaponifiable (IS). The eluting solvents for NF, GLs, and PLs were chloroform, acetone, and methanol, respectively. Solvents were evaporated with a rotary evaporator, and the percentage of each fraction was calculated as described previously [56,57,58,59,60,61,62,63,64].

### 4.5. Analysis of N. sativa Total Oil Lipid Components Using GC–MS

Gas chromatography–mass spectrometry (GC–MS) analysis was used for the quantitative analysis of 18 lipid compounds of *N. sativa*. The test was performed according to the method of Dalli et al. [65].

On dry lipid extract, 1 mL of BF3 was added (boron trifluoride–methanol solution 14%). The mixture was transmethylated at 100 °C to remove nonpolar lipids. Methyl esters were extracted two times with hexane in the presence of water. Fatty acid methyl esters were analyzed using the gas chromatography–mass spectrometry electron impact ionization method (HP/MSD Agilent 5890), operating in the scan mode (*m*/*z* = 25–2000). Samples were injected into an HP-5MS capillary column (length: 30 m, inner diameter: 0.25 mm, film thickness: 0.25 μm) and eluted by helium at 0.3 mL/min with a split ratio of 1/10. Injector and detector temperatures were 240 °C. Analysis was carried out with an ionization energy of 70 eV (filament temperature: 150 °C, current: 600 mA, potential PM: 600 V).

Fatty acid methyl esters were identified by their retention times in comparison to those of commercial standard data bank provided by the Chemstation software (NIST 2002 and Wiley version 7.0).

### 4.6. Superoxide Anion Radical (O_2_•^−^) Scavenging Assay

The ability of total oil and its fractions to scavenge superoxide anions produced with the xanthine/xanthine oxidoreductase (XOR) system was evaluated according to Robak and Gryglewski’s method (1988) [63].

Reaction medium was composed of xanthine (100 µM) and cytochrome c (25 µM) prepared in phosphate buffer saturated with oxygen (50 mM Na_2_HPO_4_/NaH_2_PO_4_, pH 7.4) and different concentrations of total oil and its fractions. The reaction was started with the addition of XOR enzyme. After one minute, the reduced cytochrome c was detected spectrophotometrically at 550 nm. The percentage of reduced cytochrome c was determined using the following relationship:

Inhibition % = (AC − AS/AC) × 100

AC: Absorbance of control.
AS: Absorbance of samples.

### 4.7. Hydroxyl Radical (OH•) Scavenging Assay

This experiment was carried out using the Smirnoff and Cumbes method (1989) [64]. The final volume of the reaction medium was 3.0 mL, and contained 1.0 mL FeSO_4_ (1.5 mM), 0.7 mL hydrogen peroxide (6 mM), 0.3 mL salicylate sodium (20 mM), and 1 mL of different concentrations of total oil and its fractions. After incubation at 37 °C for 1 h, salicylate hydroxyl complex absorption was measured at 562 nm. The percentage of OH• inhibition was calculated using the following formula:

Inhibition % = [1 − (A1 − A2)/A0] × 100

A0: Absorbance of control (without extract).
A1: Absorbance of extracts.

### 4.8. Hydrogen Peroxide (H_2_O_2_) Scavenging Assay

The ability of TO and its fractions to scavenge H_2_O_2_ was determined according to Ruch et al. [65].

H_2_O_2_ solution (40 mM) was prepared in phosphate buffer (pH 7.4). The reaction mixture consisted of 1.2 mL of H_2_O_2_ solution (40 mM in phosphate buffer, pH 7.4) and 2 mL of extracts or standard. Absorbance was measured after 10 min at 230 nm. The percentage of H_2_O_2_ scavenging was calculated using the following formula:

Inhibition % = (AC − AE)/AC × 100

AC: Absorbance of control.
AE: Absorbance of samples

### 4.9. Cell Viability Assay

Cell viability was evaluated using a 3-(4,5-dimethylthiazol-2-yl)- 2,5-diphenyl-2H- tetrazolium bromide (MTT) assay [66]. *N. sativa* TO and its fractions NF, GLs, PLs, and unsaponifiable (IS) were dissolved in DMSO (0.01%). For each fraction, two standard concentrations were made, i.e., 25 and 50 µg/mL from the initial stock solution prepared at a concentration of 100 mg of extract/1 mL of dimethyl sulfoxide (DMSO).

This test was conducted using 24-well microplates. ASM cells were incubated with the medium for one week to ensure their growth, washed with PBS, and serum-starved using DMEM supplemented with antibiotics, 4 mML-glutamine, 20 mM HEPES (pH 7.4), and 0.1% BSA. ASM cells were then incubated with different concentrations of TO and their respective fractions NF, GLs, PLs, and IS (25 and 50 µg/mL) for 24 and 48 h. The medium was aspirated and 200 μL of a medium consisting of 5% of fetal bovine serum (FBS) and 0.5 mg/mL of MTT solvent was added to each well for four hours. Following the incubation, cell supernatants were removed and replaced with 200 μL of dimethyl sulfoxide (DMSO). Basal was treated with vehicle only and DMSO. The absorbance was read at 570 nm by an ELISA reader, and the comparison was conducted against the basal.

### 4.10. Measurement of the Different CCL5, CXCL-10, and CXCL-8 in Human ASM Cell Supernatants

All ELISA assays for CCL5, CXCL-10, and CXCL-8 were performed using Duoset ELISAs (R&D Systems, Minneapolis, MN, USA), as previously reported [52].

Cultured ASM cells were treated in duplicate with NF, GLs, PLs, and IS at two concentrations of 25 and 50 µg/mL for two hours, followed by treatment with recombinant human TNF-α (10 ng/mL) and IFN-γ (25 ng/mL) (R&D Systems) for 24 h before supernatants were collected for chemokine assays using ELISA [52,67]. The light absorbance was recorded at a wavelength of 450 nm for the three chemokines.

### 4.11. Statistical Analysis

The comparison between multiple groups was made using one-way ANOVA followed by the Bonferroni post hoc test for comparisons between specific groups. The comparison between two groups was made using paired or unpaired *t*-tests, and *p* < 0.05 was taken as statistically significant.

## 5. Conclusions

The present study demonstrates that *N. sativa* TO, NF, GL, PL, and IS fractions differentially change the production of chemokines known to play a key role in the pathogenesis of asthma. We also show that *N. sativa* has radical scavenging activity. These results are still insufficient to claim that *N. sativa* has anti-inflammatory properties. In vivo data should be provided. Understanding how these different fractions exert an effect on GC-insensitive features in ASM cells may offer new therapeutic options for asthma.

## Figures and Tables

**Table 1:** Yield of *N. sativa* oil fractions.

Fractions	Yield (g/mL)	Yield (%)
Total oil (TO)	11.79	13.61 of seeds weight
Neutral lipids (NLs)	10.08	93.97 of TO
Glycolipids (GLs)	0.35	2.98 of TO
Phospholipids (FLs)	0.13	1.11 of TO

**Table 2:** Fatty acids composition of *N. sativa* total oil.

Retention Time (min)	Components	Content (%)
14.44	o-Cymen	3.5
15.15	Gamma-Terpinene	1.04
15.82	p-Cymene	0.19
16.54	m-Cymene	0.19
19.32	Alpha-Longipinene	0.16
21.67	(+)-Longifolene	1.59
22.95	Squalene	0.53
16.25	Cumene	0.12
16.92	1-Ethyl-3,5-Dimethyl-Benzene	0.51
18.59	Prehnitol	0.38
22.62	(E)5-Octadecene	0.75
23.64	Heptadecane	1.04
23.94	Not identified	26.21
30.74	Sandaracopimaradiene	3.28
29.89	Palmitic acid	8.81
32.51	Stearic acid	4.09
32.90	Oleic acid	22.58
33.72	Linoleic acid	25.03

**Table 3:** Comparison of total oil fatty acid contents of *Nigella ativa* L. from Algeria, Egypt, India, and Ethiopia [32].

Fatty Acid Composition	Total Oil Content (%)			
	Algerian	Egyptian	Indian	Ethiopian
Oleic acid	22.58	24.7	19.09	17.63
Linoleic acid	25.03	51.8	50.24	61.25
Palmitic acid	8.81	18.4	10.83	11.36
Stearic acid	4.09	2.07	2.47	2.81

## Data Availability

Not applicable.

## References

[1] Caramori G., Nucera F., Mumby S., Lo Bello F., Adcock I.M. (2022). Corticosteroid resistance in asthma: Cellular and molecular mechanisms. Mol. Asp. Med..

[2] Amrani Y., Panettieri R.A., Ramos-Ramirez P., Schaafsma D., Kaczmarek K., Tliba O. (2020). Important lessons learned from studies on the pharmacology of glucocorticoids in human airway smooth muscle cells: Too much of a good thing may be a problem. Pharmacol. Ther..

[3] Chachi L., Gavrila A., Tliba O., Amrani Y. (2015). Abnormal corticosteroid signalling in airway smooth muscle: Mechanisms and perspectives for the treatment of severe asthma. Clin. Exp. Allergy.

[4] Albano G.D., Gagliardo R.P., Montalbano A.M., Profita M. (2022). Overview of the Mechanisms of Oxidative Stress: Impact in Inflammation of the Airway Diseases. Antioxidants.

[5] Saunders R.M., Biddle M., Amrani Y., Brightling C.E. (2022). Stressed out—The role of oxidative stress in airway smooth muscle dysfunction in asthma and COPD. Free. Radic. Biol. Med..

[6] Rahman I. (2002). Oxidative stress and gene transcription in asthma and chronic obstructive pulmonary disease: Antioxidant therapeutic targets. Curr. Drug Targets-Inflamm. Allergy.

[7] Tenscher K., Metzner B., Schöpf E., Norgauer J., Czech W. (1996). Recombinant human eotaxin induces oxygen radical production, Ca^2+^-mobilization, actin reorganization, and CD11b upregulation in human eosinophils via a pertussis toxin-sensitive heterotrimeric guanine nucleotide-binding protein. Blood.

[8] Chihara J., Hayashi N., Kakazu T., Yamamoto T., Kurachi D., Nakajima S. (1994). RANTES augments radical oxygen products from eosinophils. Int. Arch. Allergy Immunol..

[9] Zhang J., Wang X., Vikash V., Ye Q., Wu D., Liu Y., Dong W. (2016). ROS and ROS-mediated cellular signaling. Oxidative Med. Cell. Longev..

[10] Sies H., Jones D.P. (2020). Reactive oxygen species (ROS) as pleiotropic physiological signalling agents. Nat. Rev. Mol. Cell Biol..

[11] Michaeloudes C., Abubakar-Waziri H., Lakhdar R., Raby K., Dixey P., Adcock I.M., Mumby S., Bhavsar P.K., Chung K.F. (2022). Molecular mechanisms of oxidative stress in asthma. Mol. Asp. Med..

[12] Torequl Islam M. (2016). Biological activities and therapeutic promises of *Nigella sativa* L.. Int. J. Pharma Sci. Sci. Res..

[13] Randhawa M.A., Alghamdi M.S. (2002). A review of the pharmacotherapeutic effects of *Nigella sativa*. Pak. J. Med. Res..

[14] Ghaznavi K.M. (1991). Tibbe-e-Nabvi aur Jadid Science, Al-Faisal Nasheeran wa Tajeera-e-Kutab.

[15] Ahlatci A., Kuzhan A., Taysi S., Demirtas O.C., Alkis H.E., Tarakcioglu M., Demirci A., Caglayan D., Saricicek E., Cinar K. (2014). Radiation-modifying abilities of *Nigella sativa* and thymoquinone on radiation-induced nitrosative stress in the brain tissue. Phytomedicine.

[16] Sharma P.C., Yelne M.B., Dennis T.J. (2005). Database on Medicinal Plants Used in Ayurveda.

[17] Khare C.P. (2004). Encyclopedia of Indian Medicinal Plants.

[18] Mahdavi R., Namazi N., Alizadeh M., Farajnia S. (2016). *Nigella sativa* oil with a calorie-restricted diet can improve biomarkers of systemic inflammation in obese women: A randomized double-blind, placebo-controlled clinical trial. J. Clin. Lipidol..

[19] Al-Attass S.A., Zahran F.M., Turkistany S.A. (2016). *Nigella sativa* and its active constituent thymoquinone in oral health. Saudi Med. J..

[20] Ahmad A., Husain A., Mujeeb M., Khan S.A., Najmi A.K., Siddique N.A., Zoheir A.D., Firoz A. (2013). A review on therapeutic potential of *Nigella sativa*: A miracle herb. Asian Pac. J. Trop. Biomed..

[21] Ahmad S., Beg Z.H. (2016). Evaluation of therapeutic effect of omega-6 linoleic acid and thymoquinone enriched extracts from *Nigella sativa* oil in the mitigation of lipidemic oxidative stress in rats. Nutrition.

[22] Hajhashemi V., Ghannadi A., Jafarabadi H. (2004). Black cumin seed essential oil, as a potent analgesic and anti-inflammatory drug. Phytother. Res..

[23] Koshak A., Wei L., Koshak E., Wali S., Alamoudi O., Demerdash A., Qutub M., Pushparaj P.N., Heinrich M. (2017). *Nigella sativa* Supplementation Improves Asthma Control and Biomarkers: A Randomized, Double-Blind, Placebo-Controlled Trial. Phytother. Res..

[24] Salem A.M., Bamosa A.O., Qutub H.O., Gupta R.K., Badar A., Elnour A., Afzal M.N. (2017). Effect of *Nigella sativa* supplementation on lung function and inflammatory mediatorsin partly controlled asthma: A randomized controlled trial. Ann. Saudi Med..

[25] Salem M.L. (2005). Immunomodulatory and therapeutic properties of the *Nigella sativa* L. seed. Int. Immunopharmacol..

[26] Kalus U., Pruss A., Bystron J., Jurecka M., Smekalova A., Lichius J.J., Kiesewetter H. (2003). Effect of *Nigella sativa* (black seed) on subjective feeling in patients with allergic diseases. Phytother. Res..

[27] Boskabady M., Shahabi M. (1997). Bronchodilatory and anticholinergic effects of *Nigella sativa* on isolated guinea-pig tracheal chains. Iran. J. Med. Sci..

[28] Boskabady M., Shirmohammadi B. (2002). Effect of *Nigella sativa* on isolated guinea pig trachea. Arch Iran. Med..

[29] Boskabady M.H., Shirmohammadi B., Jandaghi P., Kiani S. (2004). Possible mechanism(s) for relaxant effect of aqueous and macerated extracts from *Nigella sativa* on tracheal chains of guinea pig. BMC Pharmacol..

[30] Boskabady M.H., Mohsenpoor N., Takaloo L. (2010). Antiasthmatic effect of *Nigella sativa* in airways of asthmatic patients. Phytomedicine.

[31] Ramadan M.F., Kroh L.W., Mörsel J.T. (2003). Radical scavenging activity of black cumin (*Nigella sativa* L.), coriander (*Coriandrumsativum* L.), and niger (*Guizotia abyssinica* Cass.) crude seed oils and oil fractions. J. Agric. Food Chem..

[32] Thilakarathna R.C.N., Madhusankha G.D.M.P., Navaratne S.B. (2018). Morphological characteristics of black cumin (*Nigella sativa*) seeds. Chem. Res. J..

[33] Bonesi R., Saab M., Tenuta A.M., Leporini M.C., Saab M., Loizzo M.J., Tundis M.R. (2020). Screening of traditional Lebanese medicinal plants as antioxidants and inhibitors of key enzymes linked to type 2 diabetes. Plant Biosyst..

[34] Babar Z.M., Azizi W.M., Ichwan S.J.A., Ahmed Q.U., Azad A.K., Mawa I. (2019). A simple method for extracting both active oily and water soluble extract (WSE) from *Nigella sativa* (L.) seeds using a single solvent system. Nat. Prod. Res..

[35] Dalli M., Bekkouch O., Azizi S.-e., Azghar A., Gseyra N., Kim B. (2022). *Nigella sativa* L. Phytochemistry and Pharmacological Activities: A Review (2019–2021). Biomolecules.

[36] Afifa Z.B., Meriem J., Mansour Z., Asma A.Z., Hichem B.J. (2016). Physico-chemical properties, composition and antioxidant activityof seed oil from the Tunisian virginia creeper (*Parthenocissus quinquefolia* (L.) planch). J. Tunis. Chem. Soc..

[37] Kumar P.P., Kumaravel S., Lalitha C. (2010). Screening of antioxidant activity, total phenolics and GC-MS study of Vitexnegundo. Afr. J. Biochem. Res..

[38] Sugiura H., Ichinose M. (2008). Oxidative and nitrative stress in bronchial asthma. Antioxid. Redox Signal..

[39] Riedl M.A., Nel A.E. (2008). Importance of oxidative stress in the pathogenesis and treatment of asthma. Curr. Opin. Allergy Clin. Immunol..

[40] Fusco R., Cordaro M., Siracusa R., Peritore A.F., Gugliandolo E., Genovese T., D’Amico R., Crupi R., Smeriglio A., Mandalari G. (2020). Consumption of *Anacardium occidentale* L. (Cashew Nuts) Inhibits Oxidative Stress through Modulation of the Nrf2/HO−1 and NF-kB Pathways. Molecules.

[41] Saadat S., Aslani M.R., Ghorani V., Keyhanmanesh R., Boskabady M.H. (2021). The effects of *Nigella sativa* on respiratory, allergic and immunologic disorders, evidence from experimental and clinical studies, a comprehensive and updated review. Phytother. Res..

[42] Oliver B.G., Black J.L. (2006). Airway smooth muscle and asthma. Allergol. Int..

[43] Chung K.F. (2005). The role of airway smooth muscle in the pathogenesis of airway wall remodeling in chronic obstructive pulmonary disease. Proc. Am. Thorac. Soc..

[44] Tliba O., Amrani Y. (2008). Airway smooth muscle cell as an inflammatory cell: Lessons learned from interferon signaling pathways. Proc. Am. Thorac. Soc..

[45] Halayko A.J., Amrani Y. (2003). Mechanisms of inflammation-mediated airway smooth muscle plasticity and airways remodeling in asthma. Respir. Physiol. Neurobiol..

[46] John M., Hirst S.J., Jose P.J., Robichaud A., Berkman N., Witt C., Twort C.H., Barnes P.J., Chung K.F. (1997). Human airway smooth muscle cells express and release RANTES in response to T helper 1 cytokines: Regulation by T helper 2 cytokines and corticosteroids. J. Immunol..

[47] Boskabady M.H., Keyhanmanesh R., Saadatloo M.A. (2008). Relaxant effects of different fractions from *Nigella sativa* L. on guinea pig tracheal chains and its possible mechanism(s). Indian J. Exp. Biol..

[48] Boskabady M., Kiani S., Jandaghi P. (2004). Stimulatory effect of *Nigella sativa* on Β2-adrenoceptors of guinea pig tracheal chains. Med. J. Islam. Repub. Iran (MJIRI).

[49] Maier E., Duschl A., Horejs-Hoeck J. (2012). STAT6-dependent and –independent mechanisms in Th2 polarization. Eur. J. Immunol..

[50] Barlianto W., Rachmawati M., Irawan M., Wulandari D. (2017). Effects of *Nigella sativa* oil on Th1/Th2, cytokine balance, and improvement of asthma control. Paediatr. Indones..

[51] Chachi L., Abbasian M., Gavrila A., Alzahrani A., Tliba O., Bradding P., Wardlaw A.J., Brightling C., Amrani Y. (2017). Protein phosphatase 5 mediates corticosteroid insensitivity in airway smooth muscle in patients with severe asthma. Allergy.

[52] Chachi L., Shikotra A., Duffy S.M., Tliba O., Brightling C., Bradding P., Amrani Y. (2013). Functional KCa3.1 channels regulate steroid insensitivity in bronchial smooth muscle cells. J. Immunol..

[53] Ramadan M.F., Mörsel J. (2002). Direct isocratic normal-phase HPLC assay of fat-soluble vitamins and β-carotene in oilseeds. Eur. Food Res. Technol..

[54] Nieto G., Martínez L., Castillo J., Ros G. (2017). Effect of hydroxytyrosol, walnut and olive oil on nutritional profile of Low-Fat Chicken Frankfurters. Eur. J. Lipid Sci. Technol..

[55] Martínez L., Ros G., Nieto G. (2018). Fe, Zn and Se Bioavailability in Chicken Meat Emulsions Enriched with Minerals, Hydroxytyrosol and Extra Virgin Olive Oil as Measured by Caco-2 Cell Model. Nutrients.

[56] Martínez G.N. (2013). Incorporation of by-products of rosemary and thyme in the diet of ewes: Effect on the fatty acid profile of lamb. Eur. Food Res. Technol..

[57] Martínez L., Ros G., Nieto G. (2020). Effect of natural extracts obtained from food industry by-products on nutritional quality and shelf life of chicken nuggets enriched with organic Zn and Se provided in broiler diet. Poult. Sci..

[58] Nieto G., Bañón S., Garrido M. (2012). Administration of distillate thyme leaves into the diet of Segureña ewes: Effect on lamb meat quality. Animal.

[59] Macho-González A., Garcimartín A., López-Oliva M.E., Bastida S., Benedí J., Ros G., Nieto G., Sánchez-Muniz F.J. (2020). Can meat and meat-products induce oxidative stress?. Antioxidants.

[60] Nieto G., Bañón S., Garrido M.D. (2012). Incorporation of thyme leaves in the diet of pregnant and lactating ewes: Effect on the fattyacid profile of lamb. Small Rumin. Res..

[61] Munekata P.E.S., Nieto G., Pateiro M., Lorenzo J.M. (2020). Phenolic compounds obtained from olea europaea by-products and their use to improve the quality and shelf life of meat and meat products—A review. Antioxidants.

[62] Dalli M., Daoudi N.E., Azizi S.E., Benouda H., Bnouham M., Gseyra N. (2021). Chemical Composition Analysis Using HPLC-UV/GC-MS and Inhibitory Activity of Different *Nigella sativa* Fractions on Pancreatic α-Amylase and Intestinal Glucose Absorption. Biomed. Res. Int..

[63] Robak J., Gryglewski R.J. (1988). Flavonoids are scavengers of superoxide anions. Biochem. Pharmacol..

[64] Smirnoff N., Cumbes Q.J. (1989). Hydroxyl radical scavenging activity of compatible solutes. Phytochemistry.

[65] Ruch R.J., Cheng S.J., Klaunig J.E. (1989). Prevention of cytotoxicity and inhibition of intercellular communication by antioxidant catechins isolated from Chinese green tea. Carcinogenesis.

[66] Stoev S., Denev S., Dutton M., Nkosi B. (2009). Cytotoxic effect of some mycotoxins and their combinations on human peripheral blood mononuclear cells as measured by the MTT assay. Open Toxinology J..

[67] Tliba O., Panettieri R.A., Tliba S., Walseth T.F., Amrani Y. (2004). Tumor necrosis factor-alpha differentially regulates the expression of proinflammatory genes in human airway smooth muscle cells by activation of interferon-beta-dependent CD38 pathway. Mol. Pharmacol..

